# *catena*-Poly[[tetra­aqua­[μ_2_-1,4-bis­(1,2,4-triazol-1-yl)butane-κ^2^ *N* ^4^:*N* ^4′^]cadmium(II)] sulfate]

**DOI:** 10.1107/S1600536810045009

**Published:** 2010-11-10

**Authors:** Jing-Jing Song, Kou-Lin Zhang, Seik Weng Ng

**Affiliations:** a College of Chemistry and Chemical Engineering, Yangzhou University, Yangzhou 225002, People’s Republic of China; b Department of Chemistry, University of Malaya, 50603 Kuala Lumpur, Malaysia

## Abstract

In the polymeric title compound, {[Cd(C_8_H_12_N_6_)(H_2_O)_4_]SO_4_}_*n*_, the Cd^II^ atom is located on an inversion center and coordinated by four water mol­ecules and two 1,4-bis­(1,2,4-triazol-yl)butane ligands in a distorted CdO_4_N_2_ octa­hedral geometry. The 1,4-bis­(1,2,4-triazol-yl)butane ligand is centrosymmetric, the mid-point of the central C—C bond being located on an inversion center. It links adjacent water-coordinated metal atoms into polymeric chains running along the *c* axis. Adjacent chains are linked by O—H⋯N hydrogen bonds. The S atom of the sulfate anion is located on a twofold rotation axis, thus the sulfate anion is equally disordered over two sites. The sulfate anion links with the polymeric chains *via* O—H⋯O hydrogen bonds, generating a three-dimensional supra­molecular network.

## Related literature

For a related structure, see: Ding *et al.* (2008 ▶).
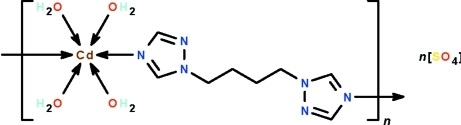


## Experimental

### 

#### Crystal data

[Cd(C_8_H_12_N_6_)(H_2_O)_4_]SO_4_
*M* *_r_* = 472.76
Monoclinic, 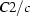

*a* = 12.1858 (9) Å
*b* = 10.9733 (8) Å
*c* = 12.4916 (9) Å
β = 90.227 (1)°
*V* = 1670.3 (2) Å^3^
*Z* = 4
Mo *K*α radiation
μ = 1.48 mm^−1^
*T* = 295 K
0.35 × 0.20 × 0.10 mm

#### Data collection

Bruker SMART APEX diffractometer
Absorption correction: multi-scan (*SADABS*; Sheldrick, 1996 ▶) *T*_min_ = 0.625, *T*_max_ = 0.866
7091 measured reflections
1922 independent reflections
1703 reflections with *I* > 2σ(*I*)
*R*_int_ = 0.026

#### Refinement

*R*[*F* ^2^ > 2σ(*F* ^2^)] = 0.024
*wR*(*F* ^2^) = 0.075
*S* = 1.03
1922 reflections
145 parameters
25 restraints
H atoms treated by a mixture of independent and constrained refinement
Δρ_max_ = 0.57 e Å^−3^
Δρ_min_ = −0.86 e Å^−3^

### 

Data collection: *APEX2* (Bruker, 2005 ▶); cell refinement: *SAINT* (Bruker, 2005 ▶); data reduction: *SAINT*; program(s) used to solve structure: *SHELXS97* (Sheldrick, 2008 ▶); program(s) used to refine structure: *SHELXL97* (Sheldrick, 2008 ▶); molecular graphics: *X-SEED* (Barbour, 2001 ▶); software used to prepare material for publication: *publCIF* (Westrip, 2010 ▶).

## Supplementary Material

Crystal structure: contains datablocks global, I. DOI: 10.1107/S1600536810045009/xu5081sup1.cif

Structure factors: contains datablocks I. DOI: 10.1107/S1600536810045009/xu5081Isup2.hkl

Additional supplementary materials:  crystallographic information; 3D view; checkCIF report

## Figures and Tables

**Table 1:** Selected bond lengths (Å)

Cd1—O1	2.3308 (18)
Cd1—O2	2.2923 (19)
Cd1—N1	2.297 (2)

**Table 2:** Hydrogen-bond geometry (Å, °)

*D*—H⋯*A*	*D*—H	H⋯*A*	*D*⋯*A*	*D*—H⋯*A*
O1—H11⋯O3	0.84 (3)	1.94 (2)	2.765 (10)	169 (4)
O1—H12⋯N2^i^	0.84 (3)	2.04 (1)	2.855 (3)	165 (4)
O2—H21⋯O4	0.84 (3)	1.91 (1)	2.736 (4)	167 (4)
O2—H21⋯O5^ii^	0.84 (3)	1.79 (2)	2.588 (4)	158 (4)
O2—H22⋯O4^iii^	0.83 (3)	1.94 (2)	2.749 (4)	162 (4)
O2—H22⋯O6^iv^	0.83 (3)	1.99 (2)	2.753 (4)	152 (4)

Symmetry codes: (i) 
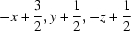
; (ii) 
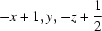
; (iii) 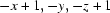; (iv) 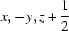.

**Fig. 1.**
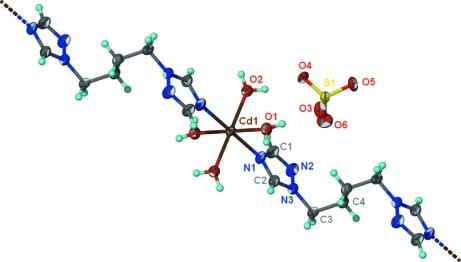
[Thermal ellipsoid plot (Barbour, 2001) of a fragment of the polymeric structure of [Cd(H2O)4(C8H12N6)2+.SO42–]n at the 50% probability level; hydrogen atoms are drawn as spheres of arbitrary radius. Inversion symmetry-related atoms are not labeled.

## supplementary crystallographic information

### Comment

The flexible bis-1,4-(1,2,4-triazol-1-yl)butane ligand binds to a number of cadmium salts to render chain motifs; when the counterion is also capable of bridging, two- and three-dimensional coordination networks are formed. The cadmium atom in polymeric [Cd(H_2_O)_4_(C_8_H_12_N_6_)^2+.^SO_4_^2–^]*_n_* (Scheme I, Fig. 1) lies on a center-of-inversion. The ligand links adjacent water-coordinated metal atoms into a chain; the sulfate ion is not directly involved in coordination to the metal center. Adjacent chains are linked by hydrogen bonds to the disordered sulfate ion to generate a three-dimensional hydrogen-bonded network (Table 1). The metal center shows octahedral coordination.

With cadmium bis(perchlorate) and bis(tetrafluoroborate), the cadmium atom is connected to two ligands, and the six-coordinate geometry is completed by two water molecules (Ding *et al.*, 2008).

### Experimental

Cadmium sulfate (0.209 g, 0.10 mmol) was dissolved in a water-DFM mixture (5 ml:3 ml), and to this was addded 1,4-bis(1,2,4-triazol-1-yl)butane (0.384 g, 0.20 mmol) dissolved in water (5 ml). The solution was set aside for the growth of colorless crystals.

### Refinement

Carbon-bound H-atoms were placed in calculated positions (C—H 0.93 Å) and were included in the refinement in the riding model approximation, with *U*(H) set to 1.2*U*(C).

The water H-atoms were located in a difference Fourier map, and were refined with a distance restraint of O–H 0.84±0.01 Å; their temperature factors were refined.

The sulfate ion is disodered with respect to the oxygen atoms only; these were refined as half-occupancy atoms off the twofold rotation axis. The sulfur–oxygen distances were restrained to within 0.01 Å of each other as were the oxygen–oxygen distances.

### Crystal data

**Table:**

[Cd(C_8_H_12_N_6_)(H_2_O)_4_]SO_4_	*F*(000) = 952
*M**_r_* = 472.76	*D*_x_ = 1.880 Mg m^−^^3^
Monoclinic, *C*2/*c*	Mo *K*α radiation, λ = 0.71073 Å
Hall symbol: -C 2yc	Cell parameters from 4284 reflections
*a* = 12.1858 (9) Å	θ = 2.5–27.6°
*b* = 10.9733 (8) Å	µ = 1.48 mm^−^^1^
*c* = 12.4916 (9) Å	*T* = 295 K
β = 90.227 (1)°	Prism, colorless
*V* = 1670.3 (2) Å^3^	0.35 × 0.20 × 0.10 mm
*Z* = 4	

### Data collection

**Table:**

Bruker SMART APEX diffractometer	1922 independent reflections
Radiation source: fine-focus sealed tube	1703 reflections with *I* > 2σ(*I*)
graphite	*R*_int_ = 0.026
ω scans	θ_max_ = 27.5°, θ_min_ = 2.5°
Absorption correction: multi-scan (*SADABS*; Sheldrick, 1996)	*h* = −15→15
*T*_min_ = 0.625, *T*_max_ = 0.866	*k* = −13→14
7091 measured reflections	*l* = −16→16

### Refinement

**Table:**

Refinement on *F*^2^	Primary atom site location: structure-invariant direct methods
Least-squares matrix: full	Secondary atom site location: difference Fourier map
*R*[*F*^2^ > 2σ(*F*^2^)] = 0.024	Hydrogen site location: inferred from neighbouring sites
*wR*(*F*^2^) = 0.075	H atoms treated by a mixture of independent and constrained refinement
*S* = 1.03	*w* = 1/[σ^2^(*F*_o_^2^) + (0.0459*P*)^2^ + 1.6989*P*] where *P* = (*F*_o_^2^ + 2*F*_c_^2^)/3
1922 reflections	(Δ/σ)_max_ = 0.001
145 parameters	Δρ_max_ = 0.57 e Å^−^^3^
25 restraints	Δρ_min_ = −0.86 e Å^−^^3^

### Fractional atomic coordinates and isotropic or equivalent isotropic displacement parameters (Å^2^)

**Table:**

	*x*	*y*	*z*	*U*_iso_*/*U*_eq_	Occ. (<1)
Cd1	0.7500	0.2500	0.5000	0.02963 (11)	
S1	0.5000	0.07764 (7)	0.2500	0.02981 (18)	
O1	0.60887 (16)	0.34194 (16)	0.40281 (15)	0.0409 (4)	
O2	0.65422 (17)	0.07057 (19)	0.50971 (16)	0.0459 (5)	
O3	0.4869 (9)	0.2093 (3)	0.2565 (9)	0.0469 (19)	0.50
O4	0.5016 (3)	0.0222 (4)	0.3542 (3)	0.0521 (10)	0.50
O5	0.4146 (3)	0.0243 (4)	0.1823 (3)	0.0525 (11)	0.50
O6	0.6087 (3)	0.0513 (4)	0.1970 (3)	0.0590 (11)	0.50
N1	0.82340 (17)	0.1877 (2)	0.34004 (15)	0.0371 (4)	
N2	0.8666 (2)	0.0739 (2)	0.19781 (18)	0.0438 (5)	
N3	0.87109 (16)	0.1946 (2)	0.17182 (16)	0.0347 (4)	
C1	0.8375 (2)	0.0763 (3)	0.2994 (2)	0.0443 (6)	
H1	0.8276	0.0059	0.3396	0.053*	
C2	0.8456 (3)	0.2611 (2)	0.2566 (2)	0.0368 (6)	
H2	0.8433	0.3458	0.2583	0.044*	
C3	0.8972 (2)	0.2345 (2)	0.0628 (2)	0.0393 (6)	
H3A	0.9610	0.1902	0.0376	0.047*	
H3B	0.9154	0.3205	0.0638	0.047*	
C4	0.8012 (2)	0.2132 (3)	−0.0145 (2)	0.0382 (5)	
H4A	0.7828	0.1272	−0.0147	0.046*	
H4B	0.8239	0.2350	−0.0864	0.046*	
H11	0.579 (3)	0.296 (3)	0.358 (2)	0.064 (10)*	
H12	0.619 (3)	0.4147 (14)	0.385 (3)	0.062 (10)*	
H21	0.616 (3)	0.056 (4)	0.455 (2)	0.078 (12)*	
H22	0.618 (3)	0.040 (4)	0.560 (2)	0.088 (14)*	

### Atomic displacement parameters (Å^2^)

**Table:**

	*U*^11^	*U*^22^	*U*^33^	*U*^12^	*U*^13^	*U*^23^
Cd1	0.03598 (16)	0.03235 (16)	0.02057 (15)	−0.00111 (8)	0.00354 (10)	−0.00067 (8)
S1	0.0366 (4)	0.0257 (4)	0.0271 (4)	0.000	−0.0052 (3)	0.000
O1	0.0520 (10)	0.0321 (10)	0.0386 (9)	−0.0044 (8)	−0.0095 (8)	0.0052 (8)
O2	0.0569 (12)	0.0455 (11)	0.0352 (10)	−0.0168 (9)	−0.0035 (9)	0.0039 (8)
O3	0.063 (5)	0.0271 (13)	0.051 (3)	0.011 (3)	−0.015 (3)	−0.009 (3)
O4	0.060 (2)	0.068 (3)	0.0287 (18)	−0.019 (2)	−0.0095 (17)	0.0215 (18)
O5	0.065 (3)	0.047 (2)	0.045 (2)	−0.019 (2)	−0.028 (2)	0.0080 (18)
O6	0.054 (2)	0.064 (3)	0.059 (3)	0.005 (2)	0.017 (2)	−0.012 (2)
N1	0.0476 (11)	0.0398 (12)	0.0240 (9)	0.0033 (9)	0.0033 (8)	−0.0036 (8)
N2	0.0611 (14)	0.0348 (11)	0.0357 (11)	0.0066 (10)	0.0042 (10)	−0.0007 (9)
N3	0.0382 (10)	0.0387 (11)	0.0273 (10)	−0.0016 (9)	0.0004 (8)	−0.0009 (9)
C1	0.0628 (16)	0.0365 (13)	0.0336 (12)	−0.0015 (12)	0.0049 (12)	0.0030 (10)
C2	0.0476 (15)	0.0340 (13)	0.0287 (13)	−0.0014 (9)	0.0025 (11)	−0.0054 (9)
C3	0.0392 (13)	0.0501 (16)	0.0287 (13)	−0.0042 (10)	0.0063 (11)	0.0036 (10)
C4	0.0464 (14)	0.0427 (13)	0.0254 (11)	−0.0018 (12)	0.0024 (10)	−0.0020 (11)

### Geometric parameters (Å, °)

**Table:**

Cd1—O1	2.3308 (18)	N1—C1	1.334 (3)
Cd1—O1^i^	2.3308 (18)	N1—C2	1.345 (4)
Cd1—O2^i^	2.2923 (19)	N2—C1	1.319 (3)
Cd1—O2	2.2923 (19)	N2—N3	1.365 (3)
Cd1—N1	2.297 (2)	N3—C2	1.325 (4)
Cd1—N1^i^	2.2967 (19)	N3—C3	1.467 (4)
S1—O4	1.437 (3)	C1—H1	0.9300
S1—O3	1.456 (3)	C2—H2	0.9300
S1—O5	1.461 (3)	C3—C4	1.533 (4)
S1—O6	1.511 (3)	C3—H3A	0.9700
O1—H11	0.84 (3)	C3—H3B	0.9700
O1—H12	0.84 (3)	C4—C4^ii^	1.530 (5)
O2—H21	0.84 (3)	C4—H4A	0.9700
O2—H22	0.83 (3)	C4—H4B	0.9700
			
O2^i^—Cd1—O2	180.0	H21—O2—H22	104 (4)
O2^i^—Cd1—N1	90.55 (8)	C1—N1—C2	103.1 (2)
O2—Cd1—N1	89.45 (8)	C1—N1—Cd1	130.93 (17)
O2^i^—Cd1—N1^i^	89.45 (8)	C2—N1—Cd1	125.14 (17)
O2—Cd1—N1^i^	90.55 (8)	C1—N2—N3	102.7 (2)
N1—Cd1—N1^i^	180.0	C2—N3—N2	109.6 (2)
O2^i^—Cd1—O1	88.59 (7)	C2—N3—C3	129.1 (2)
O2—Cd1—O1	91.41 (7)	N2—N3—C3	121.3 (2)
N1—Cd1—O1	87.98 (7)	N2—C1—N1	114.9 (2)
N1^i^—Cd1—O1	92.02 (7)	N2—C1—H1	122.6
O2^i^—Cd1—O1^i^	91.41 (7)	N1—C1—H1	122.6
O2—Cd1—O1^i^	88.59 (7)	N3—C2—N1	109.8 (2)
N1—Cd1—O1^i^	92.02 (7)	N3—C2—H2	125.1
N1^i^—Cd1—O1^i^	87.98 (7)	N1—C2—H2	125.1
O1—Cd1—O1^i^	180.0	N3—C3—C4	111.8 (2)
O4—S1—O3	111.7 (4)	N3—C3—H3A	109.3
O4—S1—O5	111.2 (2)	C4—C3—H3A	109.3
O3—S1—O5	110.6 (4)	N3—C3—H3B	109.3
O4—S1—O6	107.9 (2)	C4—C3—H3B	109.3
O3—S1—O6	108.1 (4)	H3A—C3—H3B	107.9
O5—S1—O6	107.1 (2)	C4^ii^—C4—C3	113.0 (3)
Cd1—O1—H11	114 (3)	C4^ii^—C4—H4A	109.0
Cd1—O1—H12	116 (2)	C3—C4—H4A	109.0
H11—O1—H12	118 (4)	C4^ii^—C4—H4B	109.0
Cd1—O2—H21	114 (3)	C3—C4—H4B	109.0
Cd1—O2—H22	131 (3)	H4A—C4—H4B	107.8
			
O2^i^—Cd1—N1—C1	156.6 (2)	N3—N2—C1—N1	−0.1 (3)
O2—Cd1—N1—C1	−23.4 (2)	C2—N1—C1—N2	0.0 (3)
O1—Cd1—N1—C1	−114.8 (2)	Cd1—N1—C1—N2	169.75 (18)
O1^i^—Cd1—N1—C1	65.2 (2)	N2—N3—C2—N1	−0.2 (3)
O2^i^—Cd1—N1—C2	−35.6 (2)	C3—N3—C2—N1	177.6 (2)
O2—Cd1—N1—C2	144.4 (2)	C1—N1—C2—N3	0.1 (3)
O1—Cd1—N1—C2	52.9 (2)	Cd1—N1—C2—N3	−170.44 (16)
O1^i^—Cd1—N1—C2	−127.1 (2)	C2—N3—C3—C4	−103.8 (3)
C1—N2—N3—C2	0.1 (3)	N2—N3—C3—C4	73.7 (3)
C1—N2—N3—C3	−177.9 (2)	N3—C3—C4—C4^ii^	63.0 (4)

Symmetry codes: (i) −*x*+3/2, −*y*+1/2, −*z*+1; (ii) −*x*+3/2, −*y*+1/2, −*z*.

### Hydrogen-bond geometry (Å, °)

**Table:**

*D*—H···*A*	*D*—H	H···*A*	*D*···*A*	*D*—H···*A*
O1—H11···O3	0.84 (3)	1.94 (2)	2.765 (10)	169 (4)
O1—H12···N2^iii^	0.84 (3)	2.04 (1)	2.855 (3)	165 (4)
O2—H21···O4	0.84 (3)	1.91 (1)	2.736 (4)	167 (4)
O2—H21···O5^iv^	0.84 (3)	1.79 (2)	2.588 (4)	158 (4)
O2—H22···O4^v^	0.83 (3)	1.94 (2)	2.749 (4)	162 (4)
O2—H22···O6^vi^	0.83 (3)	1.99 (2)	2.753 (4)	152 (4)

Symmetry codes: (iii) −*x*+3/2, *y*+1/2, −*z*+1/2; (iv) −*x*+1, *y*, −*z*+1/2; (v) −*x*+1, −*y*, −*z*+1; (vi) *x*, −*y*, *z*+1/2.
